# Sharing Government Health Data With the Private Sector: Community Attitudes Survey

**DOI:** 10.2196/24200

**Published:** 2021-10-01

**Authors:** Annette Braunack-Mayer, Belinda Fabrianesi, Jackie Street, Pauline O'Shaughnessy, Stacy M Carter, Lina Engelen, Lucy Carolan, Rebecca Bosward, David Roder, Kylie Sproston

**Affiliations:** 1 Australian Centre for Health Engagement, Evidence and Values School of Health and Society University of Wollongong Wollongong Australia; 2 School of Mathematics and Applied Statistics University of Wollongong Wollongong Australia; 3 School of Health and Society University of Wollongong Wollongong Australia; 4 University of South Australia Adelaide Australia; 5 Bellberry Limited South Australia Australia

**Keywords:** big data, health information systems, health data, private sector, data linkage, public opinion, consent, trust, public interest, social license

## Abstract

**Background:**

The use of government health data for secondary purposes, such as monitoring the quality of hospital services, researching the health needs of populations, and testing how well new treatments work, is increasing. This increase in the secondary uses of health data has led to increased interest in what the public thinks about data sharing, in particular, the possibilities of sharing with the private sector for research and development. Although international evidence demonstrates broad public support for the secondary use of health data, this support does not extend to sharing health data with the private sector. If governments intend to share health data with the private sector, knowing what the public thinks will be important. This paper reports a national survey to explore public attitudes in Australia toward sharing health data with private companies for research on and development of therapeutic drugs and medical devices.

**Objective:**

This study aims to explore public attitudes in Australia toward sharing government health data with the private sector.

**Methods:**

A web-based survey tool was developed to assess attitudes about sharing government health data with the private sector. A market research company was employed to administer the web-based survey in June 2019.

**Results:**

The survey was completed by 2537 individuals residing in Australia. Between 51.8% and 57.98% of all participants were willing to share their data, with slightly fewer in favor of sharing to improve health services (51.99%) and a slightly higher proportion in favor of sharing for research and development (57.98%). There was a preference for opt-in consent (53.44%) and broad support for placing conditions on sharing health information with private companies (62% to 91.99%). Wide variability was also observed in participants’ views about the extent to which the private sector could be trusted and how well they would behave if entrusted with people’s health information. In their qualitative responses, the participants noted concerns about private sector corporate interests, corruption, and profit making and expressed doubt about the Australian government’s capacity to manage data sharing safely. The percentages presented are adjusted against the Australian population.

**Conclusions:**

This nationally representative survey provides preliminary evidence that Australians are uncertain about sharing their health data with the private sector. Although just over half of all the respondents supported sharing health data with the private sector, there was also strong support for strict conditions on sharing data and for opt-in consent and significant concerns about how well the private sector would manage government health data. Addressing public concern about sharing government health data with the private sector will require more and better engagement to build community understanding about how agencies can collect, share, protect, and use their personal data.

## Introduction

### Background

Every day, people produce large amounts of health information about themselves through their interactions with health professionals, hospitals, and other government and nongovernment agencies. Beyond being a record of their health care, this information can be collated for a wide range of *secondary* uses, such as monitoring the quality of hospital services, researching the health needs of populations, and testing how well new treatments work.

As the secondary use of health data increases, so does the interest in what the public thinks about such data sharing [1-5]. This interest is related in part to growing public awareness of the risks associated with secondary use of health data, accentuated through recent data breaches and public controversies [6-10]. These events call attention to the fragility of public trust in the institutions that collect, hold, and use health data and highlight the need to understand what health data sharing the public will support, under what circumstances, for what purposes, and with whom.

Evidence from systematic and narrative reviews demonstrates broad public support for the secondary use of health data, particularly for health research [11-15]. However, research also shows that this support may not extend to sharing health data with the private sector, particularly if there is scope for commercial gain from such use [2,4,16-18]. The risks related to sharing health data, such as the potential for privacy violations, inaccuracy, misuse, discrimination, reputational damage, and embarrassment, are generally regarded as greater when sharing data with the private sector, even if it is for the purposes of research and development.

Public reticence about sharing health data with the private sector does not seem to be matched by similar concerns among governments. Rather, sharing health data with the private sector has become a component of many governments’ health and economic strategies [19-21]. For example, the use of large public data sets to support all stages of therapeutic development is one area of focus in Australia’s 2016 National Research Infrastructure Roadmap [22]. Internationally, many other countries have made similar moves through regulatory changes to increase access to and use of large public data sets [19,20]. If governments intend to share health data with the private sector, it is essential to know what the public considers important.

### Aim

This paper reports a national survey that aimed to explore public attitudes in Australia toward sharing health data with private companies for research on and development of therapeutic drugs and medical devices.

## Methods

### Ethics Approval

This study was approved by the University of Wollongong Ethics Committee. All participants provided consent before participating in the study.

### Survey Instrument

To develop the survey, we carried out an extensive review of the literature and identified demographic and sociocultural factors that might influence how the public view sharing personal health information with the private sector. We searched the peer-reviewed literature for tools to measure public attitudes toward data sharing. We developed a new instrument by combining questions from pre-existing tools with new questions and drawing on insights from the literature [16,23].

We used Survey Monkey (Momentive Inc) software to design a web-based version of the instrument [24]. To support instrument readability, the survey was piloted with a convenience sample of the general population (n=10) aged ≥14 years. We selected pilot participants to provide a diverse group with respect to age, gender, education, ethnicity, and the presence or absence of long-term illness. These participants provided feedback on the meaning of each question, the design and layout as a whole, and how long it took to complete the survey. We then refined the survey instrument, with the final survey taking approximately 9 minutes to complete. The survey was then programmed by McNair yellowSquares on the Web Survey Creator survey platform and checked for usability and technical functionality before launching.

The 11-page survey instrument included a half-page summary explaining the concepts of data linkage and sharing, including potential benefits and risks. We mentioned research and development of new drugs and medical devices and ended the introduction with the following statement: “We would like to know what you think about sharing this information with private companies such as drug companies and medical device manufacturers where the goal is to support the development of new treatments for diseases and disabilities.”

This was followed by a 29-item instrument covering sociodemographic and health-related information about participants; support for sharing health information with private companies; general views about private companies; and experience with health data collection, consent, and conditions on sharing (the survey instrument is provided in Multimedia Appendix 1). A single open-ended question at the end of the survey invited additional comments. To help participants understand that each question referred to *deidentified* government health data, the following banner appeared at the top of each page: “The questions below are about your government health information which has personal information removed, e.g. no name, no address, no date of birth, no Medicare number.”

### Recruitment and Procedures

An experienced market research company, McNair yellowSquares, recruited an opt-in sample of 2500 participants drawn from its online panel. McNair yellowSquares was asked to secure a sample that would be nationally representative by age, gender, and location. The company purposively selected participants from its panel to meet this requirement. Although potentially less ideal than probability sampling, this methodology had the practical advantage of ease of implementation and was considered appropriate for this exploratory study [25].

Australian participants of the online panel were emailed an invitation to participate in the closed survey via a unique one-time use link. Once the survey was completed, the link was disabled to prevent duplicates and the panel was regularly checked for duplication with various data points. The survey was not advertised in any manner. Up to 2 reminder emails were sent over the 3-week period during which the survey was open (May 17, 2019 to June 7, 2019). Upon completing the introductory section to establish the quotas, participants were directed to the participant information sheet, which described the researchers, purpose of the study, risks and benefits, time needed for completion, and data protection and storage. Participants were asked to indicate that they understood the participant information sheet; on assenting to this, they were directed to the first page of the survey. All questions were mandatory, and some items provided “I do not know” and “I prefer not to answer” as options. Participants were not able to view their responses by moving backward. There was no randomization of items, and all responses were captured on the McNair yellowSquares Web Survey Creator survey platform. Participation was voluntary, and participants received a small reward upon completion of all items in the survey.

McNair yellowSquares provided deidentified participant responses to the research team. All data and analyses were stored on a secure University of Wollongong server, only accessed by the research team.

### Statistical Analysis

IBM SPSS [26] was used to analyze the data. Only completed survey data were analyzed. First, we provided a descriptive summary of the survey outcomes by showing a frequency table with relative frequencies for each question of interest. The association between variables is given in cross tabulations, and *P* values are provided to answer the specific questions of interest. To support population inference, we analyzed the survey data using poststratification gender-by-age-by-state weights. We used the 2016 Australian Bureau of Statistics census data to obtain the Australian population characteristics of gender, age, and state and calculate the survey weights based on the realized sample characteristics after combining categories with small sample counts. All results except for participant demographic in this paper are obtained using the SPSS Complex Samples procedure. Raw proportions are reported to show the distributions of demographic information about the participants of this study (Table 1).

The open-ended question was analyzed inductively in NVivo (QSR International) [27]. Coding was conducted by 1 author (RB), with a second author (LC) coding half of the responses to ensure coding integrity. The authors compared coding and resolved differences before presenting the analysis to the entire research team for further discussion. The quotes in the *Results* section present examples of the diversity of responses in relation to different parts of the survey and indicate how respondents interpreted this question. A thorough analysis of this question will be presented in a separate paper.

**Table 1:** Demographics of participants (N=2537).

Characteristics			Values, n (%)
**Gender (N=2537)**			
	Male	1243 (48.99)	
	Female	1285 (50.65)	
	Other	9 (0.38)	
**Geographical area (N=2537)**			
	Metropolitan	1682 (66.29)	
	Nonmetropolitan	855 (33.7)	
**Employment (n=2526)**			
	Full-time and part-time employed	1481 (58.63)	
	Unemployed	120 (4.75)	
	Home duties	250 (9.9)	
	Student	112 (4.43)	
	Retired	456 (18.05)	
	Unable to work	107 (4.24)	
**Age (years; N=2537)**			
	<29	552 (21.76)	
	30-49	873 (34.41)	
	50-64	652 (25.7)	
	≥65	460 (18.13)	
**Highest level of education (n=2525)**			
	No formal qualifications	45 (1.80)	
	Year 10 or school certificate	265 (10.5)	
	Finished high school	422 (16.71)	
	Vocational education (trade or technical education)	840 (33.27)	
	University	953 (37.74)	
**Self-rated health (N** **=2537)**			
	Poor or fair	758 (29.88)^a^	
	Good	991 (39.06)^a^	
	Very good or excellent	788 (31.06)^a^	

^a^The Australian population—adjusted proportion.

## Results

### Participant Demographics

This data set contains responses from participants recruited by a market research company who completed the full survey. A total of 2537 participants were recruited for this study. The market research company employed to recruit participants set the location, age, and gender quota matrix to +1%. This allows for additional participants in the case of individuals being removed after data checks (eg, not located in Australia). Fewer potential participants were removed than was expected by the authors, resulting in a data set with slightly more participants than initially planned. There were equal number of male and female participants, with approximately two-third residing in metropolitan areas and 59.99% being employed. More than 20% of the participants were aged <29 years, approximately 35% were aged 30-49 years, 25% were aged 50-64 years, and 18% were aged ≥65 years. A total of 71.04% of the participants had a university or vocational qualification, and a similar proportion of the participants rated their health as good, very good, or excellent. Demographic information about the participants is provided in Table 1.

Participants were also asked if they had a My Health Record [28]. The My Health Record is a web-based summary of one’s health status, which was first launched as an opt-in record in Australia in 2012 and then controversially amended to an opt-out model in 2018. Approximately 1 in 10 Australians opted out of the system when it was amended. Just over 40.99% of participants in our survey said they had a My Health Record, 35.98% said they did not, and 23.02% were unsure.

### Sharing Health Information With Private Companies

**Figure 1:**
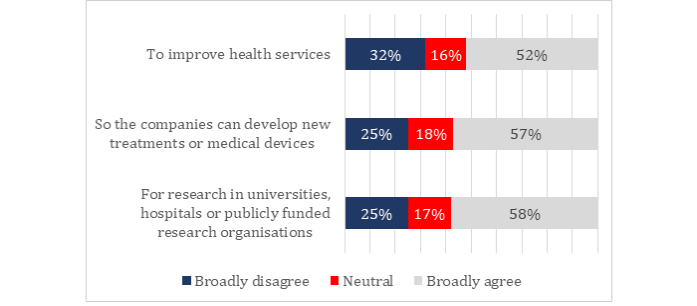
Support for sharing government health data with the private sector (N=2537): “To what extent do you agree with the government sharing your health information with private companies, such as drug companies or medical device manufactures?”.

Overall, participants were ambivalent about whether or not to share their health data with private companies. Between 50.02% and 59.99% of all participants were willing to share their data, with a slightly fewer proportion in favor of sharing to improve health services and a slightly higher proportion in favor of sharing for research and development. Figure 1 shows the degree of support for sharing health data for various purposes. The range of views was reflected in participants’ comments at the end of the survey:

I am happy to share my information if it benefits me and others.

Male, 55-59 years, metropolitan

Sharing health information with private companies is ok if the goal is to support the development of new treatments for diseases and disabilities.

Male, 18-24 years, metropolitan

I don’t think that information is any use to anybody for developing new drugs or procedures.

Male, ≥75 years, metropolitan

I don’t like my information being used by a private company.

Male, 60-64 years, metropolitan

### Conditions on Sharing

**Figure 2:**
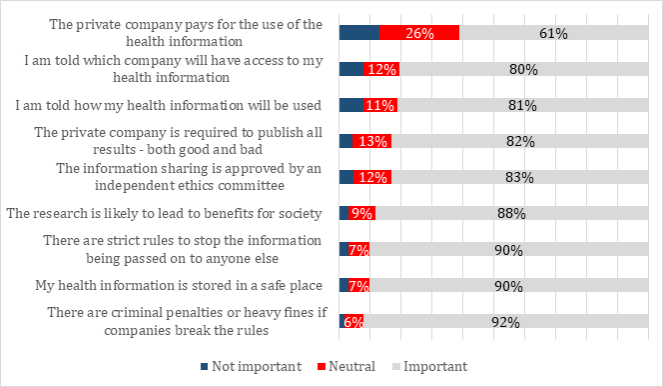
Conditions on sharing government health data with the private companies—adjusted percentages of (N=2537): “How important are various conditions if governments are to share data with private companies?”.

Participants were asked to assume that the government had decided to share their health information with a private company and to indicate the importance of various conditions on sharing. The participants responded on a scale of 1-7 with the anchors *Not important at all* and *Very important* and 4 in the neutral position. For all statements except one, 80.02% or more of the participants agreed that the condition was important. For one statement—private companies should pay for the use of the information—a small majority of participants (61.01%) considered the condition to be important. Figure 2 shows participants’ responses to the conditions on sharing government health data with private companies.

For the aforementioned conditions, we compared the responses of participants who had previously indicated that they were willing to share government health data (for all three purposes) with those who were neutral or not willing to share data (Multimedia Appendix 2). In general, participants who were willing to share data were more concerned that the conditions be met; the differences were relatively small (7.64% to 28.54%), but *P* values suggest that the differences are statistically significant. The exceptions to this finding were being told which companies would have access to health information (for all 3 purposes), how information would be used (for development and research purposes), and whether the company would pay for the data (for research purposes). For these conditions, there were no differences between the 2 groups.

### Views About Private Companies

**Figure 3:**
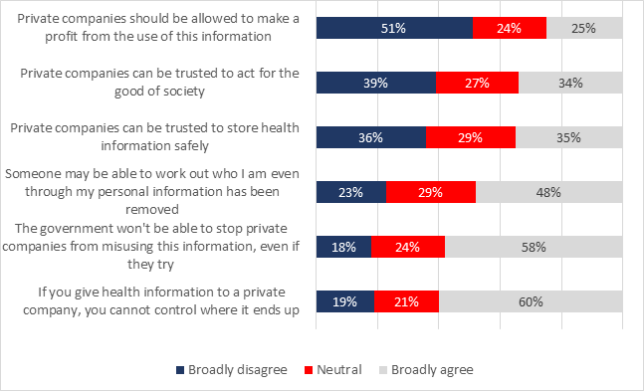
Views on sharing government health data with private companies—adjusted percentages of (N=2537): “To what extent do you agree with the following statements about private companies using government health information to support development of new treatments?”.

A series of statements were designed to assess participants’ views about what private companies could or would do if they had access to government health information (Figure 3). Participants reported their level of agreement using a 7-point Likert scale ranging from *strongly disagree* to *strongly agree*. In reporting, these have been collapsed to *broadly disagree* (1-3), *neutral* (4), and *broadly agree* (5-7). Figure 3 shows the level of broad agreement for each of the statements.

There was wide variability in participants’ responses to these statements (Figure 3). Over one-third of the participants considered that private companies could be trusted to act for the good of society or would store information safely, but these views were almost equally balanced by participants who thought the opposite. Approximately 59.99% of the participants thought that the government could not stop private companies from misusing information or control how they used it, but, again, approximately 1 out of 5 participants disagreed. Just under half of the participants said that their data could be reidentified, but at the other end of the scale, 23.02% of the participants did not think that reidentification was possible. Over half of the participants thought that private companies should not be allowed to make a profit from using the information, but one-fourth of the participants disagreed. For each statement, at least 1 in 5 participants was undecided.

The comments at the end of the survey illustrated this range of views, and concerns about corporate interests, corruption, and profit making were recurrent themes:

I think private companies will inevitably use our information for profit rather than for the greater good.

Male, 25-29 years, metropolitan

The idea of greed preventing progress and a cure is 99% of my concerns.

Female, 18-24 years, rural

I just worry that my information will not be safe.

Female, 30-34 years, metropolitan

Although this was not a survey about how the public sector held and used health data, a number of participants were equally as concerned that the government could not keep their information secure:

The current government’s record of online information processing has not been good. Look at what happened with the census.

Male, 45-49 years, metropolitan

Government is not very good at stopping anything in the past, e.g. bin full of census papers.

Male, 60-64 years, rural

We examined the relationship between the participants’ willingness to share government health data (for all 3 purposes) and their views about the private sector (Multimedia Appendix 3). The participants who had indicated that they were willing to share health data were more likely to say that private companies could be trusted (by between 51.3% and 60.82%), and that they should be able to make a profit from using government health data (51.31% to 45.62%). They were also slightly less concerned about the risk of identification (0.6% to 5.9%). Both groups of participants, who had indicated a willingness to share data and those who were neutral or did not wish to share data, were equally likely to think that any controls on data release would not work and that the government would not be able to control misuse by the private sector.

### Consent Preferences

The consent preferences of the participants are shown in Multimedia Appendix 4. There was a preference for *opt-in* consent (54.98%): it was 3 times more popular than any other option. The participants’ comments at the end of the survey reflected this view:

I would want total control over how, when and to whom my information is used and or shared with me giving the say so.

Female, 60-64 years, rural

It MUST be voluntary and OPT IN only.

Male, 65-69 years, rural

Each of the three other options for consent—refuse to share information at all, opt out, and *don’t need to know*—attracted approximately 13% of the participants. For those who wanted opt-in consent, 62.51% requested that they be asked *every time* and 23.58% wanted to give general consent and then be recontacted from time to time, whereas the rest wanted to give consent just once. Multimedia Appendix 4 shows the adjusted percentages of consent preferences.

Participants in the opt-in group were slightly more likely to rate as important the conditions that could be placed on sharing their health information than those in the opt-out group (Table 2). The largest difference was related to how their health information would be used (89.98% stating that this was important compared with 81% in the opt-out group), and the smallest difference was related to payment for use of information (62.98% compared with 59.99%).

Opt-in and opt-out participants held similar views about private companies, with differences between the 2 groups very small (2.7% to 6.6%) and nonsignificant for all but one statement (Table 3).

**Table 2:** Relationship between participants’ views on consent (opt in vs opt out) and level of agreement with the conditions on sharing data.

Conditions on sharing	Type of consent			*P* value
	Opt in (n=1356), n (%)	Opt out (n=352), n (%)		
I am told how my health information will be used	1215 (89.6)	284 (80.6)	<.001^a^	
I am told which company will have access to my health information	1189 (87.7)	284 (80.6)	.001^a^	
My health information is stored in a safe place	1285 (94.8)	312 (88.7)	<.001^a^	
The private company pays for the use of the health information	848 (62.5)	210 (59.7)	.38	
The information sharing is approved by an independent ethics committee	1187 (87.5)	286 (81.2)	.007^a^	
The private company is required to publish all results—both good and bad	1180 (87.0)	288 (81.9)	.02^a^	
The research is likely to lead to benefits for society	1246 (91.9)	304 (86.3)	.004^a^	
There are strict rules to stop the information being passed on to anyone else	1275 (94.0)	310 (88.1)	<.001^a^	

^a^Indicates level of significance at *P*<.05.

**Table 3:** Relationship between participants’ views on consent (opt in vs opt out) and views about private companies.

Views about private companies	Type of consent		*P* value
	Opt in (n=1356), n (%)	Opt out (n=352), n (%)	
Private companies can be trusted to store health information safely	494 (36.4)	119 (33.7)	.38
Private companies should be allowed to make profit from the use of this information	346 (25.5)	98 (27.8)	.41
Private companies can be trusted to act for the good of society	473 (34.9)	105 (29.9)	.10
If you give health information to a private company, you cannot control where it ends up	868 (64.0)	202 (57.4)	.04^a^
Someone may be able to work out who I am even though my personal information has been removed	679 (50.1)	157 (44.6)	.09
The government won’t be able to stop private companies from misusing this information, even if they try	818 (60.3)	199 (56.6)	.24

^a^Indicates level of significance at *P*<.05.

### Sociodemographic Patterning of Responses

We investigated the impact of various sociodemographic factors on participants’ views about whether health information should be shared and the conditions under which sharing might be acceptable (Multimedia Appendices 5-7). In general, demographic factors seemed to have only a small impact on participants’ views, with differences being less than 5.99% for most demographic factors. There were a small number of exceptions. *Older people* (aged >65 years) were more willing than the youngest age group (60.2%-70.1% compared with 49.2%-56.4%) to share their health information with private companies. They were slightly less troubled than younger people about knowing which companies would have access to their data and more committed to publishing negative results (89% compared with 78.01%). The 3 oldest age groups were more supportive of criminal penalties, and the youngest age group was least likely to agree that ethics committee oversight was needed.

Across all measures, differences between people living in *metropolitan* and *nonmetropolitan areas* were small, with the largest difference (5.01%) between the groups showing nonmetropolitan dwellers slightly less likely to support data sharing for research.

The participants’ *level of education* was related to their views, but only for some domains (Multimedia Appendices 5-7). The participants’ level of education was not related to the degree of support for sharing government health data. However, participants with higher levels of education were generally more concerned about having conditions placed on the release of data, with differences between the least and most well-educated groups ranging from 16.98% to 40.01%. For example, 81.99% of the participants with university-level education wanted ethics committee oversight of data sharing, compared with 54% for participants with only year 10-level education. Compared with participants with year 10-level education, participants with university-level education were also more likely to want to know how their information would be used (81% compared with 46.98%), which company would access their data (81% compared with 50.02%), and that all results would be published (81.98% compared with 42.01%). A history of employment in the health sector or research did not appear to influence participants’ responses.

Participants with *poorer self-reported health* *status* were slightly less likely to support (5.01% to 5.99%) sharing their health data with the private sector, as were those who *took prescribed medications* (2.99% to 5.01%). However, participants (5.01% to 7.02%) who reported *having a chronic condition* were slightly more likely to support sharing data with the private sector.

Participants who said they had a *My Health Record* were between 17.2% and 20.4% more likely than those who said they did not have a record to support sharing data with private companies for health services improvement, development, or research.

### Open-ended Question

The final question in the survey asked, “Is there anything else you would like to tell us about your views on sharing government health information with private companies where the goal is to support the development of new treatments for diseases and disabilities?” Approximately 18.01% of all respondents provided comments, primarily describing concerns about sharing government health information and the conditions under which they would support sharing or indicating support for data sharing.

Lack of trust in both private companies and the government was the most common concern. The participants cited corporate interests, corruption, and profit making as the main reasons for their distrust of private companies. They also referenced the poor track record of the government in handling data, and they questioned the ability of the government to keep their data secure and prevent misuse. Support for regulated access to health information was linked to respondents’ concerns about security:

There have been recorded cases of information being misused, be it metadata to health information. The current government’s record of online information processing has not been good. Look at what happened with the census.

Male, 45-49 years, metropolitan

Not in favour at all as I don’t trust private companies with any sort of information & same goes for this bloody lying, corrupt government!!!

Female, ≥75 years, metropolitan

I think private companies will inevitably use our information for profit rather than for the greater good.

Male, 25-29 years, metropolitan

The respondents explained that if government health information is to be shared with private companies, certain conditions need to be met. The most common requirement was anonymization of health information and a guarantee that all personal information be removed. In addition, a large subset of participants believed that data sharing needs to deliver public benefits or support the common good. They provided examples of public benefits, including developing new treatments, finding cures, or improving the health of society. Giving consent was a prerequisite to sharing health information for many participants and the right to *opt in* rather than *opt out* was highlighted by a subset.

## Discussion

### Principal Findings

This nationally representative survey provides preliminary evidence that Australians are uncertain about sharing their health data with the private sector. Although just over half of all respondents supported sharing health data with the private sector, there was also strong support for strict conditions on sharing data and for opt-in consent. These views were reinforced by participants’ ambivalence about the roles, motives, and actions of the private sector with respect to health data. Although, as a short survey, it represents relatively uninformed positions, it does indicate how people might react initially to reports of data sharing with the private sector in the news media or in public documents.

The findings of this survey demonstrate how difficult it may be to achieve policy change in this area in directions that are also acceptable to the community. Some of the conditions that participants wanted to impose on data sharing, such as using opt-in consent and providing information about each instance of use to each person who has provided data, are also conditions that some advocates of sharing would argue cannot be implemented [23,29-31]. Some intuitively attractive conditions, such as ensuring safe storage or compelling private companies to publish findings, may be difficult to enact through legislation and even more difficult to police. For example, despite decades of lament about publication bias in health research [32], relatively little headway has been made to change the practice [33-35].

A second set of challenges for policy makers may lie in identifying exactly which members of the community are concerned about what aspects of data sharing. In this survey, sociodemographic differences in views were generally small, and there were widely divergent views about what private companies could or would do if they had access to government health information. The participants who were willing to share health data were more cautious about the conditions under which they would be willing to share, but they were also more willing to trust the private sector and more willing to allow the private sector to take profits. The reasons for these findings are unclear, but they could suggest that participants had variable understandings of the private sector when answering the survey or that they had particular companies in mind. Whatever the reason, educating people about why it might be acceptable for the private sector to use public administrative data is unlikely to resonate equally across the community.

In its 2017 report on data availability and use in Australia, the Productivity Commission concluded that Australia lags behind other countries in its use of public sector data, particularly in the private sector [36]. In the Commission’s view, Australia’s foot-dragging has multiple causes, with limited community understanding and fragile trust at the top of the list, closely followed by legislative complexity, lack of leadership, data breaches, and poor data quality.

Our survey findings support the Commission’s concern about the lack of community trust in data sharing. The participants in our survey were uncertain about whether the private sector could be trusted, with at least one-third of the participants doubting the motivations and behaviors of the private sector when it came to their health information. However, many participants also agreed that sharing their health information with the private sector could yield public benefits, with just over half of all participants supporting the use of health information by private companies.

Recent scholarly studies of public views on using health data for secondary purposes also emphasize the importance of these 2 domains of trust and public benefit [15,37-39]. These recent studies cohere with our findings that understanding the benefits that can arise from using health data is necessary, but not sufficient, if the public is to entrust its health data to the private sector [10,40]. Many people are still uncomfortable with the idea of private companies accessing their government health data [41], and they have particular concerns about data privacy [42] and passing information on to marketers or insurers [43].

Building trust is not just a matter for the private sector. Trust in government is also important because it is the government that collects, holds, and releases health information in the first place [44]. This disquiet was reflected in the comments in our survey, although we did not actually ask participants to tell us their views on how well governments manage health data. Recent studies have also highlighted public misgiving about the public sector’s ability to implement and manage data sharing and linkage safely, both in general and with private companies [37,41,42]. In her article examining Australian women’s views and experiences of the My Health Record, Lupton [42] highlighted a number of well-publicized data breaches in Australia that may have contributed to participants’ cynicism about their government’s ability to keep health data protected.

At the time of this survey, we found no other Australian studies that provide a quantitative estimate of public support for sharing data with the private sector. The small number of international studies placed support for data sharing between 15% and 65% [45-47], a much larger range compared with our finding of 52% to 58%. These point estimates are helpful, but different research approaches are needed to reveal what lies beneath these numbers. Studies using focus groups, particularly in vulnerable populations; engagement and feedback through publicly focused websites; and deliberative methods such as citizens’ juries will all help explain why participants are reticent to share their health data. For some topics, the use of different methods may yield different answers. For example, we found strong support for opt-in consent in this survey, whereas deliberative studies suggest that people may become less concerned about consent when they understand that shifting to opt-in consent for the secondary use of administrative health data would make the conduct of most big data research impractical and the findings untrustworthy [16,48]. The participants in our study were probably not all that different from other people in struggling to understand how data sharing, deidentification, and data linkage work or even how administrative health data could be used for research and development.

### Limitations

This survey was conducted with an online panel of members of the public who had signed up to participate in research questionnaires, and it, therefore, has a number of limitations. In particular, as the participants were members of a panel who expressed interest and willingness to participate in research surveys, they may be more likely to be supportive of research, or at least more interested than the general public in research. The participants also probably had a reasonable level of confidence in using information technology and the internet, although what this meant for their attitudes to sharing their health data with the private sector was unclear. In addition, although participants were directed to focus on pharmaceutical companies and medical device manufacturers, it is possible that they also had other private health industries such as marketing and insurance companies in mind. This may have influenced the participants’ responses.

### Conclusions

Although there is broad public support for the secondary use of health data, our survey findings suggest that this support only extends to sharing health data with the private sector under tightly controlled circumstances. However, significant concerns are likely to remain. Addressing public concern about sharing government health data with the private sector will require more and better engagement to build community understanding about how agencies can collect, share, protect, and use their personal data.

## Appendix

Multimedia Appendix 1 Community attitudes survey.

Multimedia Appendix 2 The proportion of participants who were willing to share government health data by proportion of participants who agreed or disagreed that specific conditions should be met before sharing could occur.

Multimedia Appendix 3 The proportion of participants who were willing to share government health data by proportion of participants who agreed or disagreed on the views of private companies.

Multimedia Appendix 4 Consent preferences—adjusted percentages (N=2573): “What do you think about your health information being used by private companies for the development of new medicines or devices?”.

Multimedia Appendix 5 Adjusted percentages of willingness to share government health data with private companies by sociodemographic pattering (N=2537): “To what extent do you agree with the government sharing your health information with private companies, such as drug companies or medical device manufacturers?”.

Multimedia Appendix 6 Adjusted percentages of conditions on sharing government health data with private companies by sociodemographic patterning (N=2537): “How important is it that each of the following conditions be met when information is shared with the private sector?”.

Multimedia Appendix 7 Adjusted percentages of views on sharing government health data with private companies by sociodemographic patterning (N=2537): “To what extent do you agree with the following statements about private companies using government health information to support development of new treatments?”.
