# Supplementary material for: Sharing Government Health Data With the Private Sector: Community Attitudes Survey
Source: J Med Internet Res. 2021 Oct 1;23(10):e24200. doi: 10.2196/24200 (PMC8520136; doi:10.2196/24200)
Supplement: Multimedia Appendix 1 [file jmir_v23i10e24200_app1.pdf]

Eligibility Criteria

What is your gender?

☐	Male	☐	Indeterminate/Intersex/Trans/Gender diverse
☐	Female	☐	I prefer not to respond

How old are you?

☐	Under 18	☐	50-54
☐	18-24	☐	55-59
☐	25-29	☐	60-64
☐	30-34	☐	65-69
☐	35-39	☐	70-74
☐	40-44	☐	75+
☐	45-49		

Where do you currently live?

☐	Greater Sydney
☐	Rest of NSW
☐	Greater Melbourne
☐	Rest of VIC
☐	Greater Brisbane
☐	Rest of QLD
☐	Adelaide
☐	Rest of SA
☐	Perth
☐	Rest of WA
☐	TAS
☐	Northern Territory
☐	ACT

Linking data for development of new drugs and devices

Every day, Australians generate large amounts of information about themselves that is recorded in computers. This can include information about visits to doctors, medicines we take, hospital visits and blood tests.

Bringing together and linking these different pieces of information from lots of people provides statistics that can help us improve the quality of healthcare for all Australians. These statistics can help in the development of new treatments and make sure that the treatments we have are working and are safe. For example, by linking medical prescription and hospital emergency department statistics, researchers can discover unrecognised harms of new medicines.

At the moment health information is rarely shared and when it is, it is usually shared only between government organisations.

Although there are benefits to linking and using this information, some people are concerned about the possibility that their health information may be given to people who shouldn’t have it, or that these people may be able to work out who we are. People are also worried about private companies misusing health information.

For this reason, it is standard practice before sharing and linking health information to exclude names, addresses, dates of birth and Medicare numbers. Despite this precaution, there have been a small number of cases where re-identification has occurred.

We would like to know what you think about sharing this information with private companies such as drug companies and medical device manufacturers where the goal is to support the development of new treatments for diseases and disabilities.

The questions below are about your government health information which has personal information removed, e.g. no name, no address, no date of birth, no Medicare number.

1. To what extent do you agree with the government sharing your health information with private companies, such as drug companies or medical device manufacturers?

The government can share my health information with private companies:	I strongly disagree with this			I neither agree nor disagree with this			I strongly agree with this
(a) To improve health services	1	2	3	4	5	6	7
(b) For research in universities, hospitals or publicly funded research organisations	1	2	3	4	5	6	7
(c) So the companies can develop new treatments or medical devices (e.g. pacemakers, cataract surgery)	1	2	3	4	5	6	7

The question below is about your government health information which has personal information removed, e.g. no name, no address, no date of birth, no Medicare number.

2. What do you think about your health information being using by private companies for the development of new medicines or devices?

Question logic: 2b go to question 3. 2a, 2c, 2d, 2e or 2f go to question 4

☐	(a) My health information should not be used at all
☐	(b) I need to say 'yes' for my data to be used (opt in)
☐	(c) I need to say 'no' if I don't want my data to be used (opt out)
☐	(d) I do not need to know, just use the information
☐	(e) I am not sure/I do not know
☐	(f) I do not understand this question

The question below is about your government health information which has personal information removed, e.g. no name, no address, no date of birth, no Medicare number.

3. Would you like to be asked for your consent:

☐ (a) Every time
☐ (b) Just once
☐ (c) Get your general consent and be re-contacted from time-to-time

The questions below are about your government health information which has personal information removed, e.g. no name, no address, no date of birth, no Medicare number.

4. Imagine that the Government has decided to share your health information with a private company. The company intends to use the information to help develop a new treatment for a disease. How important is it that each of the following conditions be met before the information is shared?

How important is each of the following:	Not important at all			Neither important nor unimportant			Very important
(a) I am told how my health information will be used	1	2	3	4	5	6	7
(b) I am told which company will have access to my health information	1	2	3	4	5	6	7
(c) My health information is stored in a safe place	1	2	3	4	5	6	7
(d) The private company pays for the use of the health information	1	2	3	4	5	6	7
(e) The information sharing is approved by an independent ethics committee	1	2	3	4	5	6	7
(f) The private company is required to publish all results – both good and bad	1	2	3	4	5	6	7
(g) The research is likely to lead to benefits for society	1	2	3	4	5	6	7
(h) There are strict rules to stop the information being passed on to anyone else	1	2	3	4	5	6	7
(i) There are criminal penalties or heavy fines if companies break the rules	1	2	3	4	5	6	7

The questions below are about your government health information which has personal information removed, e.g. no name, no address, no date of birth, no Medicare number.

5. To what extent do you agree with the following statements about private companies using government health information to support development of new treatments?

	Strongly disagree			Neither agree not disagree			Strongly agree
(a) Private companies can be trusted to store health information safely	1	2	3	4	5	6	7
(b) Private companies should be allowed to make a profit from the use of this information	1	2	3	4	5	6	7
(c) Private companies can be trusted to act for the good of society	1	2	3	4	5	6	7
(d) If you give health information to a private company, you cannot control where it ends up	1	2	3	4	5	6	7
(e) Someone may be able to work out who I am even though my personal information has been removed	1	2	3	4	5	6	7
(f) The government won't be able to stop private companies from misusing this information, even if they try.	1	2	3	4	5	6	7

The question below is about your government health information which has personal information removed, e.g. no name, no address, no date of birth, no Medicare number.

6. Is there anything else you would like to tell us about your views on sharing government health information with private companies where the goal is to support the development of new treatments for diseases and disabilities?

Demographics

7. In general, how would you rate your health?

☐ My health is poor
☐ My health is fair
☐ My health is good
☐ My health is very good
☐ My health is excellent

8. About your health status:

	Yes	No	I am not sure/I do not know
(a) I have a chronic health condition			
(b) I care for someone with a chronic health condition			
(c) I take prescribed medication(s)			
(d) I have a My Health Record electronic health record			

9. Which best describes the highest educational qualification you have obtained?

☐ No formal qualifications
☐ Year 10 or school certificate
☐ Year 12 or leaving certificate
☐ Trade/apprenticeship
☐ Other TAFE/Certificate
☐ University degree/Higher degree
☐ I prefer not to answer/I am not sure

10. What best describes your current employment status?

☐ Full time employed
☐ Part-time employed
☐ Unemployed
☐ Home duties
☐ Student/Training
☐ Retired
☐ Unable to work (e.g. disability/Work Cover)
☐ I prefer not to answer/I am not sure

11. Have you worked or do you currently work in the health industry and/or in health services or research?

- [ ] Yes
- [ ] No
- [ ] I am not sure
- [ ] I prefer not to answer
