# Supplementary figures and images for: Sharing Government Health Data With the Private Sector: Community Attitudes Survey
Source: J Med Internet Res. 2021 Oct 1;23(10):e24200. doi: 10.2196/24200 (PMC8520136; doi:10.2196/24200)

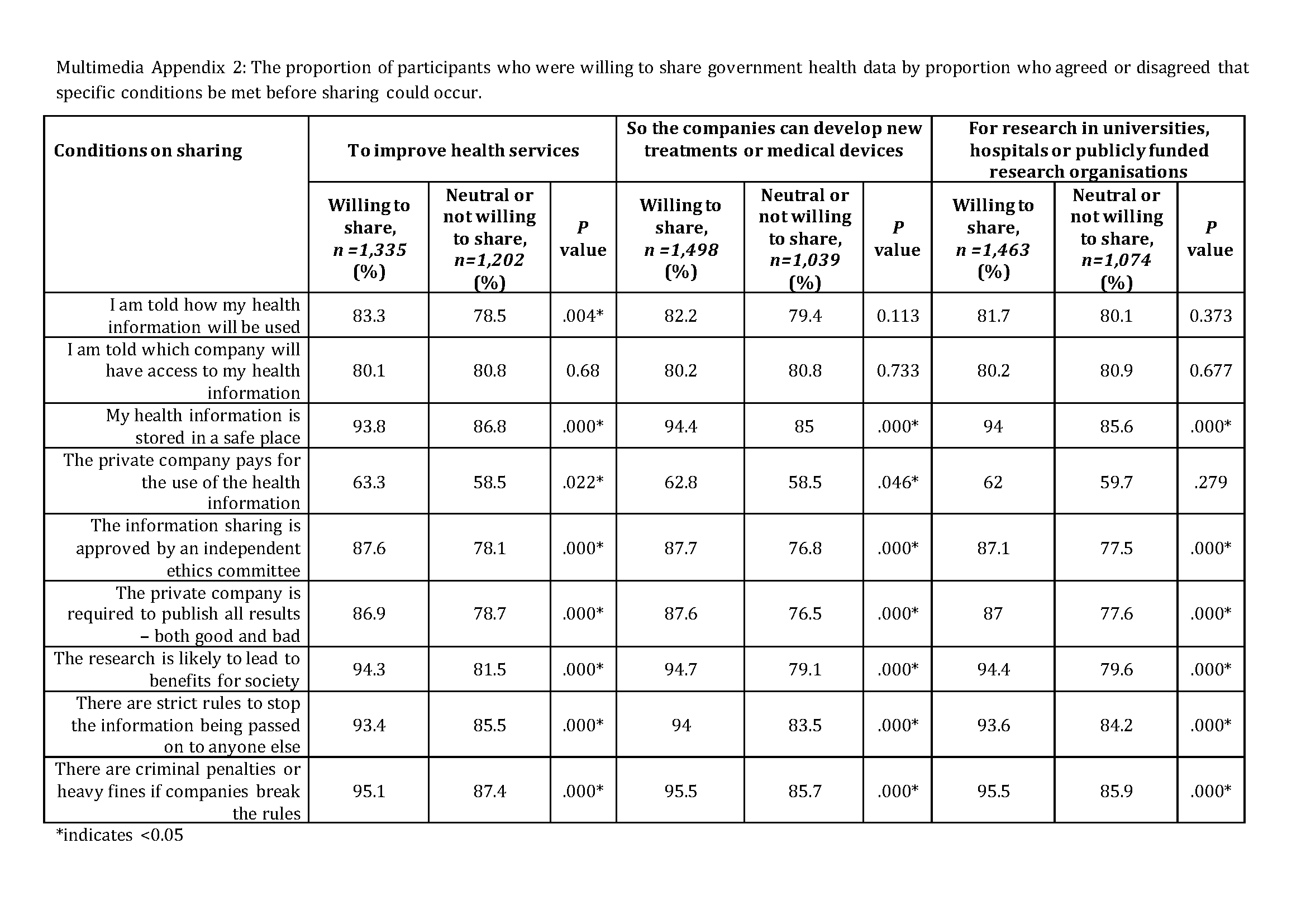

Supplement: Multimedia Appendix 2 [file jmir_v23i10e24200_app2.png]

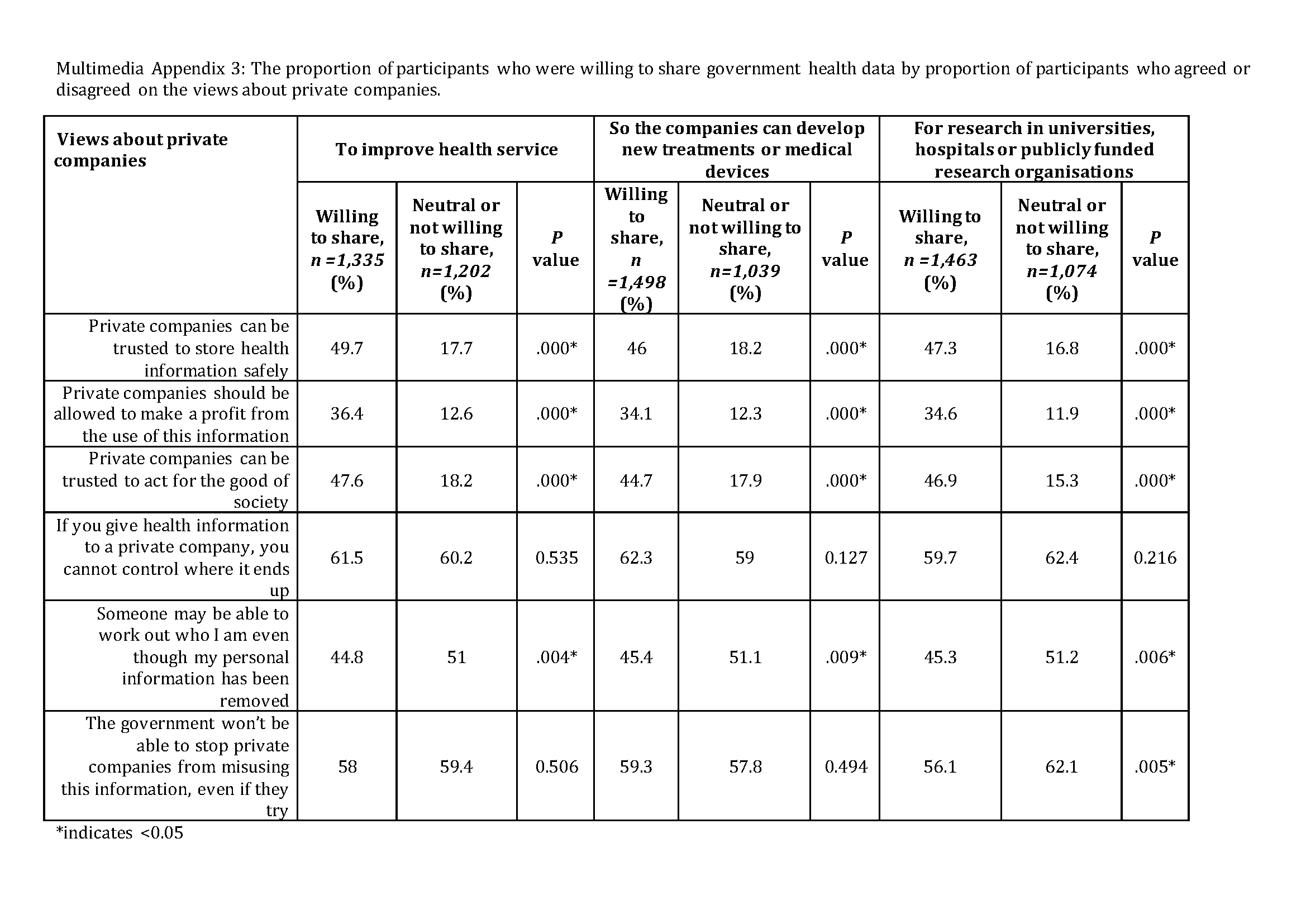

Supplement: Multimedia Appendix 3 [file jmir_v23i10e24200_app3.png]

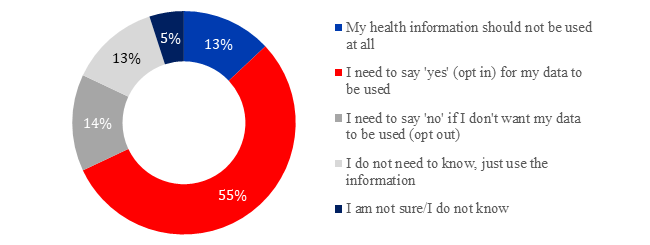

Supplement: Multimedia Appendix 4 [file jmir_v23i10e24200_app4.png]
