# Supplementary material for: Sharing Government Health Data With the Private Sector: Community Attitudes Survey
Source: J Med Internet Res. 2021 Oct 1;23(10):e24200. doi: 10.2196/24200 (PMC8520136; doi:10.2196/24200)
Supplement: Multimedia Appendix 5 [file jmir_v23i10e24200_app5.pdf]

Multimedia Appendix 5: Adjusted percentages of willingness to share government health data with private companies by sociodemographic pattering (n=2,537): 'To what extent do you agree with the government sharing your health information with private companies, such as drug companies or medical device manufacturers?'

The interpretation of the colours and shades are twofold. Dark blue indicates a large proportion in favour, while dark red indicates majority did not support a specific statement. A cell with a light shade of colour suggests that the proportion of supportive responses was around 50%.

Characteristics		Total	To improve health services	For research in universities, hospitals or publicly funded research organisations	So the companies can develop new treatments or medical devices
**Gender**	Male	1,243	55.2%	61.6%	59.8%
	Female	1,285	48.7%	54.4%	55.3%
	Other	9	34.4%	50.2%	33.0%
**Age**	<29	552	49.2%	54.1%	56.4%
	30-49	873	49.0%	53.9%	53.6%
	50-64	652	54.7%	61.2%	57.5%
	65+	460	60.2%	71.1%	67.3%
**Region**	Metro	1,682	52.9%	59.8%	57.6%
	Regional	855	49.8%	54.3%	57.2%
**Self-rated health**	My health is poor/fair	785	49.5%	55.8%	55.7%
	My health is good	991	50.3%	56.0%	56.3%
	My health is very good/excellent	788	55.6%	62.0%	60.2%
**About your health status - I have a chronic health condition**	Yes	640	56.0%	64.0%	61.9%
	No	1,749	51.0%	56.6%	56.6%
	I am not sure	148	45.9%	51.5%	51.5%
**About your health status - I care for someone with a chronic health condition**	Yes	323	50.4%	61.9%	59.0%
	No	2,155	51.8%	57.1%	56.8%
	I am not sure	59	58.4%	66.6%	69.4%
**About your health status - I take prescribed medication(s)**	Yes	1,274	53.8%	61.4%	60.4%
	No	1,230	50.4%	55.5%	55.0%
	I am not sure	33	42.0%	36.9%	54.3%
**About your health status - I have a My Health Record electronic health record**	Yes	1,039	59.3%	67.4%	68.1%
	No	913	42.1%	49.5%	47.7%
	I am not sure	585	54.7%	55.4%	55.0%
**Highest educational level**	No formal qualifications	45	60.2%	66.7%	64.5%

The interpretation of the colours and shades are twofold. Dark blue indicates a large proportion in favour, while dark red indicates majority did not support a specific statement. A cell with a light shade of colour suggests that the proportion of supportive responses was around 50%.					
Characteristics		Total	To improve health services	For research in universities, hospitals or publicly funded research organisations	So the companies can develop new treatments or medical devices
	Year 10 or school certificate	265	51.1%	52.7%	58.9%
	Year 12 or leaving certificate	422	50.4%	58.5%	57.4%
	Vocational Education	840	50.5%	57.8%	58.0%
	University degree / Higher degree	953	53.8%	59.3%	56.8%
**Employment**	Full time/part time employed	1,481	52.7%	58.2%	57.7%
	Unemployed	120	45.6%	49.6%	53.1%
	Home duties	250	42.6%	48.9%	52.9%
	Student / Training	112	49.4%	56.6%	51.4%
	Retired	456	58.4%	69.3%	65.4%
	Unable to work (e.g. disability / Work Cover)	107	53.6%	50.5%	53.7%
**Have you worked or do you currently work in the health industry and / or in health services or research?**	Yes	332	52.3%	62.7%	62.8%
	No	2,173	52.0%	57.2%	56.8%
	I am not sure	20	42.6%	66.6%	53.4%
	I prefer not to answer	12	34.7%	40.5%	41.1%
