# Supplementary material for: Sharing Government Health Data With the Private Sector: Community Attitudes Survey
Source: J Med Internet Res. 2021 Oct 1;23(10):e24200. doi: 10.2196/24200 (PMC8520136; doi:10.2196/24200)
Supplement: Multimedia Appendix 6 [file jmir_v23i10e24200_app6.pdf]

Multimedia Appendix 6: Adjusted percentages of conditions on sharing government health data with the private companies by socio demographic patterning (n=2,537): 'How important is it that each of the following conditions be met when information is shared with the private sector?'

The interpretation of the colours and shades are twofold. Dark blue indicates a large proportion in favour, while dark red indicates majority did not support a specific statement. A cell with a light shade of colour suggests that the proportion of supportive responses was around 50%.

Characteristics		Total	I am told how my health information will be used	I am told which company will have access to my health information	My health information is stored in a safe place	The private company pays for the use of the health information	The information sharing is approved by an independent ethics committee	The private company is required to publish all results - both good and bad	The research is likely to lead to benefits for society	There are strict rules to stop the information being passed on to anyone else	There are criminal penalties or heavy fines if companies break the rules
**Gender**	Male	1,243	78.6%	78.7%	88.5%	64.3%	81.8%	83.0%	86.7%	88.1%	89.6%
	Female	1,285	83.3%	82.2%	92.3%	57.6%	84.3%	83.1%	89.7%	91.1%	93.2%
	Other	9	83.4%	75.6%	83.4%	75.3%	66.7%	59.2%	66.7%	66.7%	83.4%
**Age**	<29	552	81.3%	81.3%	88.0%	60.0%	77.3%	78.4%	84.4%	85.9%	88.7%
	30-49	873	80.7%	81.3%	89.2%	63.3%	83.8%	81.8%	86.3%	88.3%	89.3%
	50-64	652	84.2%	82.3%	94.5%	63.9%	88.7%	88.3%	93.6%	95.4%	95.8%
	65+	460	76.4%	74.2%	93.5%	55.4%	88.7%	89.8%	93.9%	93.5%	96.3%
**Region**	Metro	1,682	81.7%	81.0%	90.2%	62.2%	83.3%	82.6%	88.3%	89.4%	90.7%
	Regional	855	79.7%	79.4%	91.0%	58.7%	82.4%	83.6%	87.7%	90.0%	92.6%
**Self-rated health**	My health is poor/fair	785	81.1%	80.2%	88.7%	60.0%	82.3%	84.0%	87.4%	90.2%	89.4%
	My health is good	991	79.9%	80.0%	90.3%	61.0%	83.3%	82.1%	87.2%	90.0%	91.4%

The interpretation of the colours and shades are twofold. Dark blue indicates a large proportion in favour, while dark red indicates majority did not support a specific statement. A cell with a light shade of colour suggests that the proportion of supportive responses was around 50%.

Characteristics		Total	I am told how my health information will be used	I am told which company will have access to my health information	My health information is stored in a safe place	The private company pays for the use of the health information	The information sharing is approved by an independent ethics committee	The private company is required to publish all results - both good and bad	The research is likely to lead to benefits for society	There are strict rules to stop the information being passed on to anyone else	There are criminal penalties or heavy fines if companies break the rules
	My health is very good/excellent	788	82.3%	81.2%	91.9%	61.8%	83.3%	83.1%	89.8%	88.6%	93.0%
**About your health status - I have a chronic health condition**	Yes	640	86.6%	86.7%	91.0%	71.1%	93.4%	86.6%	89.1%	88.9%	97.8%
	No	1,749	81.3%	81.6%	88.4%	58.3%	83.0%	84.5%	90.2%	89.7%	93.2%
	I am not sure	148	76.9%	76.7%	86.2%	57.6%	81.5%	80.6%	84.9%	86.8%	87.9%
**About your health status - I care for someone with a chronic**	Yes	323	80.6%	80.6%	93.4%	58.4%	82.3%	82.5%	88.6%	90.6%	92.6%
	No	2,155	83.2%	81.7%	90.4%	64.9%	84.1%	83.9%	88.9%	90.3%	91.2%
	I am not sure	59	81.1%	81.0%	90.2%	62.5%	83.8%	82.8%	87.2%	89.5%	90.4%

The interpretation of the colours and shades are twofold. Dark blue indicates a large proportion in favour, while dark red indicates majority did not support a specific statement. A cell with a light shade of colour suggests that the proportion of supportive responses was around 50%.

Characteristics		Total	I am told how my health information will be used	I am told which company will have access to my health information	My health information is stored in a safe place	The private company pays for the use of the health information	The information sharing is approved by an independent ethics committee	The private company is required to publish all results - both good and bad	The research is likely to lead to benefits for society	There are strict rules to stop the information being passed on to anyone else	There are criminal penalties or heavy fines if companies break the rules
**health condition**											
**About your health status - I take prescribed medication(s)**	Yes	1,274	79.0%	78.9%	86.5%	62.7%	64.1%	66.7%	79.5%	80.0%	84.1%
	No	1,230	84.8%	84.6%	92.1%	55.5%	78.1%	83.3%	89.3%	89.7%	92.7%
	I am not sure	33	86.8%	84.5%	90.7%	59.7%	85.5%	82.0%	89.2%	87.6%	91.4%
**About your health status - I have a My Health Record electronic health record**	Yes	1,039	76.5%	75.8%	93.1%	57.4%	88.6%	90.3%	94.3%	93.5%	95.9%
	No	913	81.6%	76.2%	86.4%	66.4%	87.4%	84.9%	88.5%	94.2%	95.6%
	I am not sure	585	80.4%	80.4%	91.3%	60.0%	87.4%	84.8%	91.1%	91.6%	94.6%

The interpretation of the colours and shades are twofold. Dark blue indicates a large proportion in favour, while dark red indicates majority did not support a specific statement. A cell with a light shade of colour suggests that the proportion of supportive responses was around 50%.

Characteristics		Total	I am told how my health information will be used	I am told which company will have access to my health information	My health information is stored in a safe place	The private company pays for the use of the health information	The information sharing is approved by an independent ethics committee	The private company is required to publish all results - both good and bad	The research is likely to lead to benefits for society	There are strict rules to stop the information being passed on to anyone else	There are criminal penalties or heavy fines if companies break the rules
**Highest educational level**	No formal qualifications	45	81.5%	80.8%	90.7%	61.1%	82.7%	83.2%	88.0%	89.7%	91.2%
	Year 10 or school certificate	265	46.6%	49.6%	64.6%	54.9%	53.8%	42.0%	69.8%	61.9%	65.3%
	Year 12 or leaving certificate	422	75.6%	82.0%	70.1%	75.6%	70.1%	64.2%	63.5%	70.1%	82.0%
	Vocational Education	840	81.0%	80.2%	91.5%	57.0%	87.3%	87.2%	91.4%	91.9%	92.8%
	University degree / Higher degree	953	80.7%	80.4%	90.4%	61.9%	81.7%	82.2%	87.2%	89.0%	91.0%
**Employment**	Full time/part	1,481	84.3%	82.4%	87.2%	65.3%	83.0%	76.8%	87.2%	88.1%	90.1%

The interpretation of the colours and shades are twofold. Dark blue indicates a large proportion in favour, while dark red indicates majority did not support a specific statement. A cell with a light shade of colour suggests that the proportion of supportive responses was around 50%.

Characteristics		Total	I am told how my health information will be used	I am told which company will have access to my health information	My health information is stored in a safe place	The private company pays for the use of the health information	The information sharing is approved by an independent ethics committee	The private company is required to publish all results - both good and bad	The research is likely to lead to benefits for society	There are strict rules to stop the information being passed on to anyone else	There are criminal penalties or heavy fines if companies break the rules
	time employed										
	Unemployed	120	76.5%	78.4%	91.0%	59.9%	83.1%	84.5%	90.2%	90.1%	88.0%
	Home duties	250	81.7%	80.6%	90.5%	60.9%	82.9%	82.9%	87.8%	89.9%	91.9%
	Student / Training	112	80.2%	85.5%	84.5%	67.7%	84.8%	78.5%	88.9%	77.1%	88.3%
	Retired	456	81.5%	79.0%	90.5%	57.3%	85.7%	85.2%	89.7%	91.6%	92.8%
	Unable to work (e.g. disability / Work Cover)	107	80.9%	81.7%	90.8%	64.0%	80.9%	81.6%	87.1%	88.4%	90.2%
**Have you worked or do you currently work in the**	Yes	332	70.2%	80.7%	76.9%	67.7%	74.8%	63.3%	74.2%	69.6%	87.0%
	No	2,173	81.6%	78.7%	92.3%	58.2%	84.3%	84.8%	92.0%	93.3%	94.5%
	I am not sure	20	81.7%	83.3%	88.4%	65.2%	82.0%	81.7%	84.1%	85.7%	88.5%
	I prefer not to answer	12	78.8%	79.0%	90.6%	59.0%	82.4%	81.8%	87.9%	89.5%	90.6%

The interpretation of the colours and shades are twofold. Dark blue indicates a large proportion in favour, while dark red indicates majority did not support a specific statement. A cell with a light shade of colour suggests that the proportion of supportive responses was around 50%.										
Characteristics	Total	I am told how my health information will be used	I am told which company will have access to my health information	My health information is stored in a safe place	The private company pays for the use of the health information	The information sharing is approved by an independent ethics committee	The private company is required to publish all results - both good and bad	The research is likely to lead to benefits for society	There are strict rules to stop the information being passed on to anyone else	There are criminal penalties or heavy fines if companies break the rules
**health industry and / or in health services or research?**										
