# Supplementary material for: Sharing Government Health Data With the Private Sector: Community Attitudes Survey
Source: J Med Internet Res. 2021 Oct 1;23(10):e24200. doi: 10.2196/24200 (PMC8520136; doi:10.2196/24200)
Supplement: Multimedia Appendix 7 [file jmir_v23i10e24200_app7.pdf]

Multimedia Appendix 7: Adjusted percentages of views on sharing government health data with the private companies by socio demographic patterning (n=2,537): 'To what extent do you agree with the following statements about private companies using government health information to support development of new treatments?'

The interpretation of the colours and shades are twofold. Dark blue indicates a large proportion in favour, while dark red indicates majority did not support a specific statement. A cell with a light shade of colour suggests that the proportion of supportive responses was around 50%.

Characteristics		Total	Private companies can be trusted to store health information safely	Private companies should be allowed to make a profit from the use of this information	Private companies can be trusted to act for the good of society	If you give health information to a private company, you cannot control where it ends up	Someone may be able to work out who I am even though my personal information has been removed	The government won't be able to stop private companies from misusing this information, even if they try
**Gender**	Male	1,243	36.4%	32.2%	38.1%	63.1%	49.7%	59.7%
	Female	1,285	32.4%	18.0%	29.0%	58.6%	46.0%	57.5%
	Other	9	16.4%	8.3%	16.4%	67.3%	49.2%	66.9%
**Age**	<29	552	37.2%	25.0%	35.8%	53.0%	47.8%	53.1%
	30-49	873	33.8%	24.2%	32.3%	61.6%	50.2%	59.9%
	50-64	652	29.4%	24.4%	29.7%	69.5%	48.3%	64.5%
	65+	460	34.4%	26.9%	34.4%	68.3%	42.6%	63.1%
**Region**	Metro	1,682	34.9%	26.4%	34.8%	60.2%	48.7%	59.3%
	Regional	855	33.2%	22.1%	30.7%	62.2%	46.0%	57.5%

The interpretation of the colours and shades are twofold. Dark blue indicates a large proportion in favour, while dark red indicates majority did not support a specific statement. A cell with a light shade of colour suggests that the proportion of supportive responses was around 50%.

Characteristics		Total	Private companies can be trusted to store health information safely	Private companies should be allowed to make a profit from the use of this information	Private companies can be trusted to act for the good of society	If you give health information to a private company, you cannot control where it ends up	Someone may be able to work out who I am even though my personal information has been removed	The government won't be able to stop private companies from misusing this information, even if they try
**Self-rated health**	My health is poor/fair	785	30.5%	22.5%	29.9%	65.8%	51.4%	63.2%
	My health is good	991	35.0%	25.3%	34.6%	61.2%	46.2%	59.2%
	My health is very good/excellent	788	36.6%	26.4%	35.0%	56.4%	46.6%	54.3%
**About your health status - I have a chronic health condition**	Yes	640	32.1%	22.4%	30.3%	68.8%	49.4%	61.5%
	No	1,749	35.7%	25.8%	34.9%	58.3%	47.3%	57.4%
	I am not sure	148	26.6%	24.1%	28.3%	60.8%	48.1%	63.0%
**About your health status - I care for someone with a chronic health condition**	Yes	323	34.3%	28.2%	35.3%	60.6%	50.8%	61.2%
	No	2,155	34.3%	24.5%	33.1%	60.9%	47.2%	58.0%
	I am not sure	59	33.7%	25.2%	35.5%	61.3%	54.7%	68.7%
	Yes	1,274	32.5%	24.9%	33.2%	65.9%	46.9%	61.3%

The interpretation of the colours and shades are twofold. Dark blue indicates a large proportion in favour, while dark red indicates majority did not support a specific statement. A cell with a light shade of colour suggests that the proportion of supportive responses was around 50%.

Characteristics		Total	Private companies can be trusted to store health information safely	Private companies should be allowed to make a profit from the use of this information	Private companies can be trusted to act for the good of society	If you give health information to a private company, you cannot control where it ends up	Someone may be able to work out who I am even though my personal information has been removed	The government won't be able to stop private companies from misusing this information, even if they try
**About your health status - I take prescribed medication(s)**	No	1,230	35.9%	24.4%	33.6%	56.5%	48.5%	56.0%
	I am not sure	33	33.4%	43.7%	35.4%	57.8%	49.8%	68.6%
**About your health status - I have a My Health Record electronic health record**	Yes	1,039	38.8%	28.1%	36.6%	61.3%	44.0%	57.7%
	No	913	31.2%	24.9%	32.2%	62.0%	55.5%	60.4%
	I am not sure	585	31.7%	19.5%	29.9%	58.2%	41.7%	57.4%
**Highest educational level**	No formal qualifications	45	33.4%	26.7%	37.9%	64.3%	57.4%	64.3%
	Year 10 or school certificate	265	30.2%	18.8%	28.6%	63.2%	47.1%	55.7%

The interpretation of the colours and shades are twofold. Dark blue indicates a large proportion in favour, while dark red indicates majority did not support a specific statement. A cell with a light shade of colour suggests that the proportion of supportive responses was around 50%.								
Characteristics		Total	Private companies can be trusted to store health information safely	Private companies should be allowed to make a profit from the use of this information	Private companies can be trusted to act for the good of society	If you give health information to a private company, you cannot control where it ends up	Someone may be able to work out who I am even though my personal information has been removed	The government won't be able to stop private companies from misusing this information, even if they try
	Year 12 or leaving certificate	422	33.9%	23.2%	31.1%	55.0%	43.8%	53.6%
	Vocational Education	840	33.8%	24.1%	34.8%	61.4%	47.1%	59.9%
	University degree / Higher degree	953	36.3%	28.2%	34.7%	63.0%	50.1%	60.9%
**Employment**	Full time/part time employed	1,481	35.8%	26.1%	35.2%	60.4%	47.5%	58.1%
	Unemployed	120	32.7%	17.2%	30.5%	61.2%	51.3%	55.1%
	Home duties	250	28.3%	20.8%	29.7%	58.6%	51.3%	57.7%
	Student / Training	112	34.7%	27.9%	31.6%	51.1%	50.3%	55.9%
	Retired	456	33.2%	27.1%	33.1%	67.1%	43.4%	63.4%
	Unable to work (e.g. disability / Work Cover)	107	33.5%	15.9%	25.8%	68.3%	49.1%	62.4%

The interpretation of the colours and shades are twofold. Dark blue indicates a large proportion in favour, while dark red indicates majority did not support a specific statement. A cell with a light shade of colour suggests that the proportion of supportive responses was around 50%.								
Characteristics		Total	Private companies can be trusted to store health information safely	Private companies should be allowed to make a profit from the use of this information	Private companies can be trusted to act for the good of society	If you give health information to a private company, you cannot control where it ends up	Someone may be able to work out who I am even though my personal information has been removed	The government won't be able to stop private companies from misusing this information, even if they try
**Have you worked or do you currently work in the health industry and / or in health services or research?**	Yes	332	35.2%	22.9%	33.5%	64.6%	51.8%	61.3%
	No	2,173	34.5%	25.1%	33.4%	60.5%	47.3%	58.2%
	I am not sure	20	19.2%	43.5%	38.4%	47.5%	37.7%	55.6%
	I prefer not to answer	12	11.3%	17.5%	23.3%	52.5%	40.8%	69.4%
